# Diethyl 2-amino-5-[(*E*)-(1-methyl-1*H*-pyrrol-2-yl)methylideneamino]thiophene-3,4-dicarboxylate

**DOI:** 10.1107/S1600536810046775

**Published:** 2010-11-20

**Authors:** Stéphane Dufresne, W. G. Skene

**Affiliations:** a Department of Chemistry, University of Montreal, CP 6128, Succ. Centre-ville, Montréal, Québec, H3C 3J7, Canada

## Abstract

The structure of the title compound, C_16_H_19_N_3_O_4_S, shows the planes described by the thio­phene and the pyrroles are twisted by 17.06 (4)°. Additionally, the structure shows the azomethine bond adopts the *E* configuration, while the pyrrole is disordered as a heterocycle flip [occupancy ratio 0.729 (5):0.271 (5)]. The three-dimensional network is well packed and involves N–H⋯O hydrogen bonding and π–π stacking [centroid–centroid distance = 4.294 (8) Å].

## Related literature

For our on-going research on conjugated azomethines, see: Dufresne & Skene (2008 ▶). For bond lengths in comparable azomethines, see: Skene *et al.* (2006 ▶); Dufresne & Skene (2010 ▶).
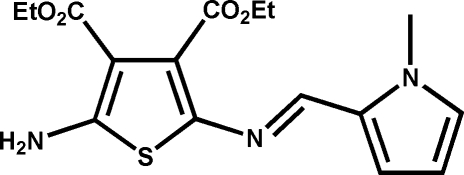


## Experimental

### 

#### Crystal data

C_16_H_19_N_3_O_4_S
*M* *_r_* = 349.40
Monoclinic, 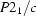

*a* = 8.8212 (18) Å
*b* = 9.0799 (18) Å
*c* = 21.793 (4) Å
β = 97.50 (3)°
*V* = 1730.6 (6) Å^3^
*Z* = 4
Cu *K*α radiation
μ = 1.89 mm^−1^
*T* = 123 K
0.17 × 0.16 × 0.15 mm

#### Data collection

Bruker SMART 6000 diffractometer
Absorption correction: multi-scan (*SADABS*; Sheldrick,1996 ▶) *T*_min_ = 0.710, *T*_max_ = 0.762
20876 measured reflections
3367 independent reflections
3046 reflections with *I* > 2σ(*I*)
*R*_int_ = 0.034

#### Refinement

*R*[*F* ^2^ > 2σ(*F* ^2^)] = 0.042
*wR*(*F* ^2^) = 0.116
*S* = 1.07
3367 reflections
267 parameters
32 restraints
H-atom parameters constrained
Δρ_max_ = 0.32 e Å^−3^
Δρ_min_ = −0.54 e Å^−3^

### 

Data collection: *SMART* (Bruker, 2003 ▶); cell refinement: *SAINT* (Bruker, 2004 ▶); data reduction: *SAINT*; program(s) used to solve structure: *SHELXS97* (Sheldrick, 2008 ▶); program(s) used to refine structure: *SHELXL97* (Sheldrick, 2008 ▶); molecular graphics: *SHELXTL* (Sheldrick, 2008 ▶) and *ORTEP-3* (Farrugia, 1997 ▶); software used to prepare material for publication: *UdMX* (Marris, 2004 ▶).

## Supplementary Material

Crystal structure: contains datablocks I, global. DOI: 10.1107/S1600536810046775/bh2321sup1.cif

Structure factors: contains datablocks I. DOI: 10.1107/S1600536810046775/bh2321Isup2.hkl

Additional supplementary materials:  crystallographic information; 3D view; checkCIF report

## Figures and Tables

**Table 1:** Hydrogen-bond geometry (Å, °)

*D*—H⋯*A*	*D*—H	H⋯*A*	*D*⋯*A*	*D*—H⋯*A*
N1—H1*B*⋯O3^i^	0.88	2.09	2.925 (3)	157

Symmetry code: (i) 
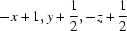
.

**Fig. 1.**
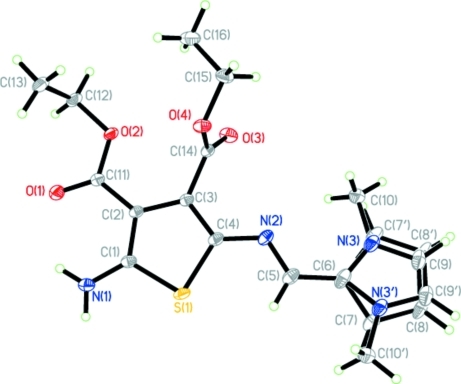
ORTEP representation of the title molecule with the numbering scheme adopted (Farrugia, 1997). The disorder on the pyrrole unit is represented by prime symbols. Ellipsoids drawn at 30% probability level.

**Fig. 2.**
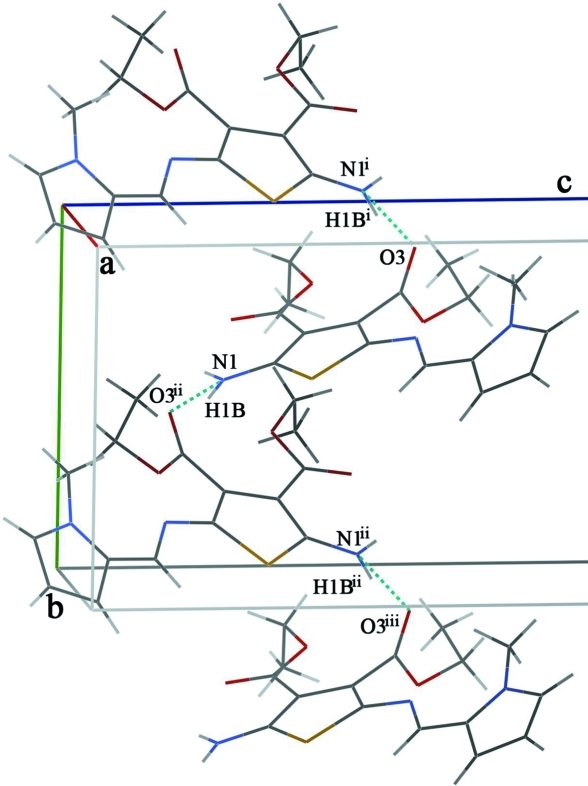
Supramolecular structure showing the intermolecular H-bonding giving the structural arrangement. Disorder has been omitted for clarity. Dashed lines indicate hydrogen bonds. [Symmetry codes: (i) 1 - x, -1/2 + y, 1/2 - z; (ii) 1 - x, 1/2 + y, 1/2 - z; (iii) x, 1 + y, z.]

**Fig. 3.**
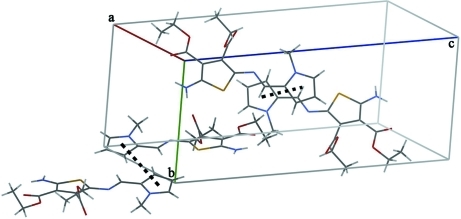
The three-dimensional network demonstrating the π-stacking in the lattice. Disorder has been omitted for clarity.

## supplementary crystallographic information

### Comment

During our on-going research relating to conjugated azomethines (Dufresne & Skene, 2008), we prepared the title compound. The structure is given in figure 1. The pyrrole is disordered. The occupation factor was found to be 73% for the *anti*periplanar heterocycle. The salient feature of the resolved structure is assigning the absolute isomer of the azomethine, which is not readily possible by other means. The *E* isomer was found and the crystal symmetry was *P*2_1_/*c*. Neither solvent nor counter-ions were found in the structure.

A major point of interest is the azomethine bond. The bond lengths for N2—C4, N2—C5 and C5—C6 are 1.372 (2), 1.292 (2) and 1.424 (2) Å, respectively. These are similar to comparable azomethines (Skene *et al.*, 2006 and Dufresne & Skene, 2010) whose homologue lengths are 1.381 (3), 1.283 (3) and 1.426 (3) Å.

We found that the heterocycles of the title compound are not coplanar, according to angle between the mean planes described by them. The angle between these planes was found to be 17.06 (4)°. This is in contrast to an analogous thiophene-azomethine compound (Skene *et al.*, 2006) whose mean plane angle is 7.25 (11)°.

Figure 2 shows the H-bonding occurring within the lattice. Only one H-bonding was found between N1—H1B···O3^ii^ with an angle of 157.1° and a distance of 2.925 (3) Å between the nitrogen and the oxygen. Hydrogen bonding and π-stacking are the driving forces for the overall assembly. π-stacking was found to take place between the pyrroles as seen in Figure 3.

### Experimental

1-Methyl-2-pyrrole-carboxaldehyde and 2,5-diamino-thiophene-3,4-dicarboxylic acid diethyl ester were mixed in anhydrous 2-propanol with a catalytic amount of TFA and refluxed for 12 h. The reaction was then purified by flash chromatography to afford the title compound as a yellow solid. Single crystals were obtained by slow evaporation of an acetone solution.

### Refinement

C-bonded H atoms were placed in calculated positions (C—H = 0.93–0.98 Å) and included in the refinement in the riding-model approximation, with *U*_iso_(H) = 1.2-1.5 *U*_eq_(C). The protons on the amino group were placed in calculated positions (N—H = 0.88 Å) and included in the refinement in the riding-model approximation, with *U*_iso_(H) = 1.2 *U*_eq_(N). During the refinement, evidence came that the structure was disordered as an inversion of terminal heterocycles. We first tried to fix each part to half of the weight and then let it vary to the optimized proportion of 73:27. We were forced to add constraints to the minor counterpart so it looks like the major one. We used fixed similar temperature factors, as well as distances and angles restraints with every disordered atom.

### Crystal data

**Table:**

C_16_H_19_N_3_O_4_S	*F*(000) = 736
*M**_r_* = 349.40	*D*_x_ = 1.341 Mg m^−^^3^
Monoclinic, *P*2_1_/*c*	Melting point: 404(2) K
Hall symbol: -P 2ybc	Cu *K*α radiation, λ = 1.54178 Å
*a* = 8.8212 (18) Å	Cell parameters from 10603 reflections
*b* = 9.0799 (18) Å	θ = 4.1–71.3°
*c* = 21.793 (4) Å	µ = 1.89 mm^−^^1^
β = 97.50 (3)°	*T* = 123 K
*V* = 1730.6 (6) Å^3^	Block, yellow
*Z* = 4	0.17 × 0.16 × 0.15 mm

### Data collection

**Table:**

Bruker SMART 6000 diffractometer	3367 independent reflections
Radiation source: Rotating Anode	3046 reflections with *I* > 2σ(*I*)
Montel 200 optics	*R*_int_ = 0.034
Detector resolution: 5.5 pixels mm^-1^	θ_max_ = 72.0°, θ_min_ = 4.1°
ω scans	*h* = −10→10
Absorption correction: multi-scan (*SADABS*; Sheldrick,1996)	*k* = −11→11
*T*_min_ = 0.710, *T*_max_ = 0.762	*l* = −26→25
20876 measured reflections	

### Refinement

**Table:**

Refinement on *F*^2^	Primary atom site location: structure-invariant direct methods
Least-squares matrix: full	Secondary atom site location: difference Fourier map
*R*[*F*^2^ > 2σ(*F*^2^)] = 0.042	Hydrogen site location: inferred from neighbouring sites
*wR*(*F*^2^) = 0.116	H-atom parameters constrained
*S* = 1.07	*w* = 1/[σ^2^(*F*_o_^2^) + (0.0845*P*)^2^ + 0.153*P*] where *P* = (*F*_o_^2^ + 2*F*_c_^2^)/3
3367 reflections	(Δ/σ)_max_ < 0.001
267 parameters	Δρ_max_ = 0.32 e Å^−^^3^
32 restraints	Δρ_min_ = −0.54 e Å^−^^3^
0 constraints	

### Fractional atomic coordinates and isotropic or equivalent isotropic displacement parameters (Å^2^)

**Table:**

	*x*	*y*	*z*	*U*_iso_*/*U*_eq_	Occ. (<1)
S1	0.45470 (4)	0.43388 (4)	0.275627 (17)	0.02979 (14)	
O1	0.80431 (13)	0.22781 (12)	0.16548 (5)	0.0338 (3)	
O2	0.89746 (11)	0.11974 (11)	0.25566 (5)	0.0280 (2)	
O3	0.73230 (12)	0.04262 (11)	0.38224 (5)	0.0347 (3)	
O4	0.89935 (11)	0.22984 (11)	0.38638 (5)	0.0288 (2)	
N1	0.56568 (15)	0.42094 (15)	0.16734 (6)	0.0352 (3)	
H1A	0.6308	0.3913	0.1426	0.042*	
H1B	0.4906	0.4809	0.1534	0.042*	
N2	0.50239 (13)	0.31869 (14)	0.39473 (6)	0.0304 (3)	
C1	0.58092 (15)	0.37474 (15)	0.22635 (6)	0.0255 (3)	
C2	0.69216 (14)	0.28057 (14)	0.25579 (6)	0.0217 (3)	
C3	0.67137 (15)	0.25520 (14)	0.31923 (6)	0.0227 (3)	
C4	0.54893 (15)	0.32748 (16)	0.33720 (7)	0.0268 (3)	
C5	0.39981 (16)	0.40771 (17)	0.41036 (8)	0.0328 (3)	
H5	0.3630	0.4830	0.3820	0.039*	
C6	0.33869 (17)	0.39978 (19)	0.46758 (8)	0.0377 (4)	
C11	0.80092 (15)	0.20966 (14)	0.22090 (6)	0.0231 (3)	
C12	1.01267 (19)	0.04672 (18)	0.22452 (8)	0.0370 (4)	
H12A	0.9660	0.0140	0.1830	0.044*	
H12B	1.0502	−0.0417	0.2484	0.044*	
C13	1.14477 (19)	0.1469 (2)	0.21800 (9)	0.0468 (5)	
H13A	1.1091	0.2309	0.1918	0.070*	
H13B	1.2228	0.0928	0.1990	0.070*	
H13C	1.1887	0.1824	0.2589	0.070*	
C14	0.76941 (15)	0.16206 (14)	0.36484 (6)	0.0229 (3)	
C15	1.00158 (18)	0.15225 (19)	0.43389 (7)	0.0357 (4)	
H15A	0.9401	0.0962	0.4608	0.043*	
H15B	1.0642	0.2248	0.4600	0.043*	
C16	1.1047 (2)	0.0488 (2)	0.40530 (9)	0.0498 (5)	
H16A	1.0429	−0.0259	0.3811	0.075*	
H16B	1.1741	0.0005	0.4380	0.075*	
H16C	1.1644	0.1040	0.3782	0.075*	
N3	0.3667 (7)	0.3033 (4)	0.5110 (3)	0.0300 (10)	0.729 (5)
C7	0.2273 (5)	0.5042 (6)	0.4841 (2)	0.0307 (9)	0.729 (5)
H7	0.1852	0.5864	0.4609	0.037*	0.729 (5)
C8	0.1955 (9)	0.4567 (9)	0.5423 (3)	0.0351 (13)	0.729 (5)
H8	0.1274	0.5019	0.5670	0.042*	0.729 (5)
C9	0.2812 (8)	0.3328 (7)	0.5569 (3)	0.0339 (12)	0.729 (5)
H9	0.2807	0.2761	0.5935	0.041*	0.729 (5)
C10	0.4701 (3)	0.1768 (3)	0.51050 (11)	0.0425 (7)	0.729 (5)
H10A	0.4547	0.1301	0.4696	0.064*	0.729 (5)
H10B	0.4484	0.1055	0.5420	0.064*	0.729 (5)
H10C	0.5763	0.2104	0.5196	0.064*	0.729 (5)
N83	0.2589 (11)	0.4768 (12)	0.5004 (4)	0.0224 (17)	0.271 (5)
C87	0.369 (2)	0.2593 (12)	0.5158 (9)	0.027 (2)	0.271 (5)
H87	0.4264	0.1731	0.5101	0.033*	0.271 (5)
C88	0.290 (2)	0.2937 (17)	0.5684 (7)	0.026 (2)	0.271 (5)
H88	0.2875	0.2365	0.6048	0.031*	0.271 (5)
C89	0.221 (2)	0.426 (2)	0.5544 (8)	0.028 (3)	0.271 (5)
H89	0.1559	0.4746	0.5792	0.033*	0.271 (5)
C90	0.2156 (6)	0.6219 (7)	0.4747 (2)	0.0302 (15)	0.271 (5)
H90A	0.3063	0.6850	0.4773	0.045*	0.271 (5)
H90B	0.1400	0.6666	0.4982	0.045*	0.271 (5)
H90C	0.1713	0.6113	0.4312	0.045*	0.271 (5)

### Atomic displacement parameters (Å^2^)

**Table:**

	*U*^11^	*U*^22^	*U*^33^	*U*^12^	*U*^13^	*U*^23^
S1	0.0183 (2)	0.0303 (2)	0.0411 (2)	0.00671 (12)	0.00480 (14)	0.00178 (13)
O1	0.0328 (6)	0.0391 (6)	0.0299 (5)	0.0073 (4)	0.0059 (4)	−0.0022 (4)
O2	0.0241 (5)	0.0257 (5)	0.0357 (5)	0.0080 (4)	0.0101 (4)	0.0037 (4)
O3	0.0277 (5)	0.0292 (5)	0.0458 (6)	−0.0060 (4)	−0.0010 (5)	0.0100 (5)
O4	0.0195 (5)	0.0273 (5)	0.0379 (6)	−0.0020 (4)	−0.0026 (4)	−0.0003 (4)
N1	0.0293 (7)	0.0422 (8)	0.0333 (7)	0.0123 (5)	0.0008 (5)	0.0051 (5)
N2	0.0206 (6)	0.0346 (7)	0.0376 (7)	−0.0001 (5)	0.0096 (5)	−0.0028 (5)
C1	0.0188 (6)	0.0236 (7)	0.0334 (7)	−0.0004 (5)	0.0009 (5)	−0.0015 (5)
C2	0.0170 (6)	0.0183 (6)	0.0296 (7)	−0.0003 (5)	0.0028 (5)	−0.0015 (5)
C3	0.0172 (6)	0.0200 (6)	0.0310 (7)	−0.0017 (5)	0.0037 (5)	−0.0005 (5)
C4	0.0181 (6)	0.0264 (7)	0.0363 (7)	0.0000 (5)	0.0053 (5)	−0.0001 (5)
C5	0.0225 (7)	0.0314 (7)	0.0461 (9)	−0.0030 (5)	0.0107 (6)	−0.0042 (6)
C6	0.0253 (8)	0.0436 (9)	0.0470 (10)	−0.0086 (7)	0.0152 (7)	−0.0144 (8)
C11	0.0198 (6)	0.0196 (6)	0.0298 (7)	−0.0015 (5)	0.0033 (5)	−0.0019 (5)
C12	0.0344 (8)	0.0303 (8)	0.0499 (9)	0.0153 (6)	0.0185 (7)	0.0049 (6)
C13	0.0295 (8)	0.0536 (11)	0.0611 (11)	0.0137 (7)	0.0200 (8)	0.0188 (9)
C14	0.0176 (6)	0.0231 (6)	0.0283 (6)	−0.0012 (5)	0.0045 (5)	−0.0016 (5)
C15	0.0284 (7)	0.0422 (8)	0.0336 (8)	0.0025 (6)	−0.0072 (6)	0.0008 (6)
C16	0.0381 (10)	0.0591 (11)	0.0499 (10)	0.0204 (8)	−0.0031 (8)	0.0037 (8)
N3	0.0230 (11)	0.032 (2)	0.0357 (16)	0.001 (2)	0.0083 (9)	−0.005 (2)
C7	0.0216 (19)	0.030 (3)	0.041 (3)	0.0043 (13)	0.0066 (15)	−0.0019 (16)
C8	0.028 (2)	0.040 (3)	0.041 (3)	−0.0014 (19)	0.015 (2)	−0.012 (2)
C9	0.0328 (18)	0.044 (4)	0.026 (2)	0.000 (3)	0.0054 (17)	0.003 (2)
C10	0.0417 (14)	0.0451 (14)	0.0425 (13)	0.0156 (11)	0.0120 (10)	0.0147 (10)
N83	0.018 (4)	0.019 (4)	0.029 (4)	0.007 (3)	−0.003 (3)	0.007 (3)
C87	0.035 (4)	0.012 (5)	0.036 (4)	−0.003 (4)	0.007 (3)	0.000 (4)
C88	0.032 (4)	0.026 (6)	0.021 (5)	0.008 (4)	0.010 (4)	0.010 (3)
C89	0.031 (7)	0.031 (8)	0.023 (4)	0.000 (5)	0.009 (4)	0.008 (4)
C90	0.030 (3)	0.033 (4)	0.029 (3)	0.008 (2)	0.006 (2)	0.008 (2)

### Geometric parameters (Å, °)

**Table:**

S1—C1	1.7301 (15)	C13—H13b	0.98
S1—C4	1.7703 (15)	C13—H13c	0.98
O1—C11	1.2232 (17)	C15—C16	1.499 (2)
O2—C11	1.3407 (16)	C15—H15a	0.99
O2—C12	1.4537 (17)	C15—H15b	0.99
O3—C14	1.2080 (17)	C16—H16a	0.98
O4—C14	1.3313 (16)	C16—H16b	0.98
O4—C15	1.4622 (17)	C16—H16c	0.98
N1—C1	1.3428 (19)	N3—C9	1.354 (7)
N1—H1a	0.88	N3—C10	1.468 (4)
N1—H1b	0.88	C7—C8	1.402 (7)
N2—C5	1.2918 (19)	C7—H7	0.95
N2—C4	1.3715 (19)	C8—C9	1.369 (5)
C1—C2	1.3933 (18)	C8—H8	0.95
C2—C3	1.4368 (18)	C9—H9	0.95
C2—C11	1.4503 (18)	C10—H10a	0.98
C3—C4	1.3643 (19)	C10—H10b	0.98
C3—C14	1.4923 (18)	C10—H10c	0.98
C5—C6	1.424 (2)	N83—C89	1.346 (15)
C5—H5	0.95	N83—C90	1.463 (8)
C6—N83	1.276 (12)	C87—C88	1.450 (18)
C6—N3	1.290 (5)	C87—H87	0.95
C6—C7	1.445 (5)	C88—C89	1.361 (11)
C6—C87	1.652 (14)	C88—H88	0.95
C12—C13	1.499 (2)	C89—H89	0.95
C12—H12a	0.99	C90—H90a	0.98
C12—H12b	0.99	C90—H90b	0.98
C13—H13a	0.98	C90—H90c	0.98
			
C1—S1—C4	91.41 (7)	O3—C14—O4	124.09 (13)
C11—O2—C12	116.43 (11)	O3—C14—C3	124.09 (12)
C14—O4—C15	116.74 (11)	O4—C14—C3	111.73 (11)
C1—N1—H1A	120	O4—C15—C16	111.07 (13)
C1—N1—H1B	120	O4—C15—H15A	109.4
H1A—N1—H1B	120	C16—C15—H15A	109.4
C5—N2—C4	120.54 (14)	O4—C15—H15B	109.4
N1—C1—C2	127.53 (13)	C16—C15—H15B	109.4
N1—C1—S1	120.36 (11)	H15A—C15—H15B	108
C2—C1—S1	112.11 (11)	C15—C16—H16A	109.5
C1—C2—C3	111.76 (12)	C15—C16—H16B	109.5
C1—C2—C11	120.36 (12)	H16A—C16—H16B	109.5
C3—C2—C11	127.55 (12)	C15—C16—H16C	109.5
C4—C3—C2	113.85 (12)	H16A—C16—H16C	109.5
C4—C3—C14	119.55 (13)	H16B—C16—H16C	109.5
C2—C3—C14	126.60 (12)	C6—N3—C9	109.6 (4)
C3—C4—N2	125.24 (13)	C6—N3—C10	125.8 (4)
C3—C4—S1	110.85 (11)	C9—N3—C10	124.5 (4)
N2—C4—S1	123.90 (11)	C8—C7—C6	104.3 (4)
N2—C5—C6	123.90 (16)	C8—C7—H7	127.9
N2—C5—H5	118.1	C6—C7—H7	127.9
C6—C5—H5	118.1	C9—C8—C7	107.0 (5)
N83—C6—N3	91.6 (4)	C9—C8—H8	126.5
N83—C6—C5	139.8 (4)	C7—C8—H8	126.5
N3—C6—C5	128.3 (2)	N3—C9—C8	109.6 (5)
N3—C6—C7	109.5 (3)	N3—C9—H9	125.2
C5—C6—C7	122.2 (2)	C8—C9—H9	125.2
N83—C6—C87	97.0 (7)	C6—N83—C89	121.2 (1)
C5—C6—C87	123.2 (6)	C6—N83—C90	114.4 (7)
C7—C6—C87	114.0 (6)	C89—N83—C90	124.4 (1)
O1—C11—O2	122.99 (12)	C88—C87—C6	106.4 (9)
O1—C11—C2	124.12 (13)	C88—C87—H87	126.8
O2—C11—C2	112.89 (11)	C6—C87—H87	126.8
O2—C12—C13	111.53 (14)	C89—C88—C87	104.90 (11)
O2—C12—H12A	109.3	C89—C88—H88	127.5
C13—C12—H12A	109.3	C87—C88—H88	127.5
O2—C12—H12B	109.3	N83—C89—C88	110.30 (13)
C13—C12—H12B	109.3	N83—C89—H89	124.9
H12A—C12—H12B	108	C88—C89—H89	124.9
C12—C13—H13A	109.5	N83—C90—H90A	109.5
C12—C13—H13B	109.5	N83—C90—H90B	109.5
H13A—C13—H13B	109.5	H90A—C90—H90B	109.5
C12—C13—H13C	109.5	N83—C90—H90C	109.5
H13A—C13—H13C	109.5	H90A—C90—H90C	109.5
H13B—C13—H13C	109.5	H90B—C90—H90C	109.5
			
C4—S1—C1—N1	179.29 (13)	C2—C3—C14—O4	77.00 (16)
C4—S1—C1—C2	−1.27 (11)	C14—O4—C15—C16	86.08 (17)
N1—C1—C2—C3	−179.63 (13)	N83—C6—N3—C9	8.3 (7)
S1—C1—C2—C3	0.98 (14)	C5—C6—N3—C9	−178.0 (4)
N1—C1—C2—C11	−5.7 (2)	C7—C6—N3—C9	0.4 (6)
S1—C1—C2—C11	174.91 (9)	C87—C6—N3—C9	−126 (7)
C1—C2—C3—C4	−0.01 (16)	N83—C6—N3—C10	−174.1 (7)
C11—C2—C3—C4	−173.40 (12)	C5—C6—N3—C10	−0.4 (7)
C1—C2—C3—C14	−179.28 (12)	C7—C6—N3—C10	178.1 (5)
C11—C2—C3—C14	7.3 (2)	C87—C6—N3—C10	52 (6)
C2—C3—C4—N2	178.30 (12)	N83—C6—C7—C8	−23.70 (19)
C14—C3—C4—N2	−2.4 (2)	N3—C6—C7—C8	0.3 (5)
C2—C3—C4—S1	−0.93 (15)	C5—C6—C7—C8	178.9 (4)
C14—C3—C4—S1	178.40 (9)	C87—C6—C7—C8	7.2 (1)
C5—N2—C4—C3	169.49 (14)	C6—C7—C8—C9	−1.0 (7)
C5—N2—C4—S1	−11.4 (2)	C6—N3—C9—C8	−1.1 (8)
C1—S1—C4—C3	1.25 (11)	C10—N3—C9—C8	−178.7 (6)
C1—S1—C4—N2	−177.99 (13)	C7—C8—C9—N3	1.3 (9)
C4—N2—C5—C6	175.94 (14)	N3—C6—N83—C89	−9.00 (14)
N2—C5—C6—N83	166.7 (8)	C5—C6—N83—C89	178.70 (11)
N2—C5—C6—N3	−3.6 (4)	C7—C6—N83—C89	148 (3)
N2—C5—C6—C7	178.1 (3)	C87—C6—N83—C89	−3.40 (15)
N2—C5—C6—C87	−10.9 (9)	N3—C6—N83—C90	170.4 (7)
C12—O2—C11—O1	2.11 (19)	C5—C6—N83—C90	−2.00 (13)
C12—O2—C11—C2	−178.63 (12)	C7—C6—N83—C90	−32.20 (15)
C1—C2—C11—O1	0.9 (2)	C87—C6—N83—C90	176.0 (1)
C3—C2—C11—O1	173.81 (13)	N83—C6—C87—C88	0.70 (15)
C1—C2—C11—O2	−178.33 (11)	N3—C6—C87—C88	47 (6)
C3—C2—C11—O2	−5.44 (19)	C5—C6—C87—C88	179.20 (11)
C11—O2—C12—C13	80.18 (17)	C7—C6—C87—C88	−9.20 (17)
C15—O4—C14—O3	0.2 (2)	C6—C87—C88—C89	2(2)
C15—O4—C14—C3	176.97 (11)	C6—N83—C89—C88	5(2)
C4—C3—C14—O3	74.56 (18)	C90—N83—C89—C88	−174.20 (14)
C2—C3—C14—O3	−106.21 (17)	C87—C88—C89—N83	−4(2)
C4—C3—C14—O4	−102.23 (14)		

### Hydrogen-bond geometry (Å, °)

**Table:**

*D*—H···*A*	*D*—H	H···*A*	*D*···*A*	*D*—H···*A*
N1—H1B···O3^i^	0.88	2.09	2.925 (3)	157

Symmetry codes: (i) −*x*+1, *y*+1/2, −*z*+1/2.
